# Spontaneous regression of secondary vitreoretinal lymphoma after diagnostic vitrectomy: case report

**DOI:** 10.1186/s12886-023-02967-5

**Published:** 2023-05-17

**Authors:** Linyang Gan, Junjie Ye

**Affiliations:** Department of Ophthalmology, Peking Union Medical College Hospital, Chinese Academy of Medical Sciences, 100730 Beijing, China

**Keywords:** Secondary vitreoretinal lymphoma, Diagnostic vitrectomy, Spontaneous regression, Gastric MALT lymphoma, Case report

## Abstract

**Purpose:**

Our purpose is to report a patient with secondary intraocular mucosa-associated lymphoid tissue (MALT) who experienced spontaneous regression after diagnostic vitrectomy.

**Methods:**

We retrospectively reviewed the clinical and imaging features of the case. Multimodal imaging, including fundus photograph, optical coherence tomography, fundus fluorescein angiography and ultrasound scan was presented.

**Results:**

A 71-year-old female presented with a subretinal lesion temporal to macula and scattered multifocal creamy lesions deep to retina in her left eye. Optical coherence tomography of the left eye showed multifocal nodular hyper-reflective signals between the Bruch’s membrane and RPE. She had a history of gastric MALT lymphoma. Diagnostic vitrectomy was performed. IL-10 level of aqueous was 187.7pg/ml. Cytology, gene rearrangement and flow cytometry of the vitreous were inconclusive. Systemic evaluation was normal. Secondary vitreoretinal MALT lymphoma was considered. Interestingly, her subretinal lesions regressed gradually without any chemotherapy. And IL-10 level of aqueous declined to 64.3pg/ml.

**Conclusions:**

Secondary vitreoretinal MALT lymphoma is extremely rare. Spontaneous regression of intraocular lymphoma does occur.

**Supplementary Information:**

The online version contains supplementary material available at 10.1186/s12886-023-02967-5.

## Introduction

Secondary intraocular lymphoma arises from a known source of systemic lymphoma and metastasizes to the eye hematogenously. It disseminates predominantly to the uvea which has rich blood supply, or to the vitreous or the retina less commonly[1]. It’s typically diffuse large B cell lymphoma(DLBCL) type [1]. The major subtype of ophthalmic lymphoma is extranodal marginal zone(EMZ) lymphoma of mucosa-associated lymphoid tissue (MALT). It usually involves the conjunctiva, lacrimal gland and orbit, with intraocular involvement being rarely reported [2]. To the best of our knowledge, only one case of vitreoretinal lymphomas(VRL) of EMZ type was reported [3]. Secondary intraocular involvement by systemic MALT lymphoma is an extremely rare condition. Spontaneous regression(SR) of malignant tumors is defined as partial or complete resolution of a tumor without treatment or with therapy believed to be inadequate for the observed response [4]. SR of lymphoma has predominantly been reported for low-grade subtypes [4]. Herein, we described a patient with gastric MALT lymphoma exhibiting secondary vitreoretinal involvement, which regressed spontaneously after diagnostic pars plana vitrectomy(PPV).

## Case description

**Fig. 1:**
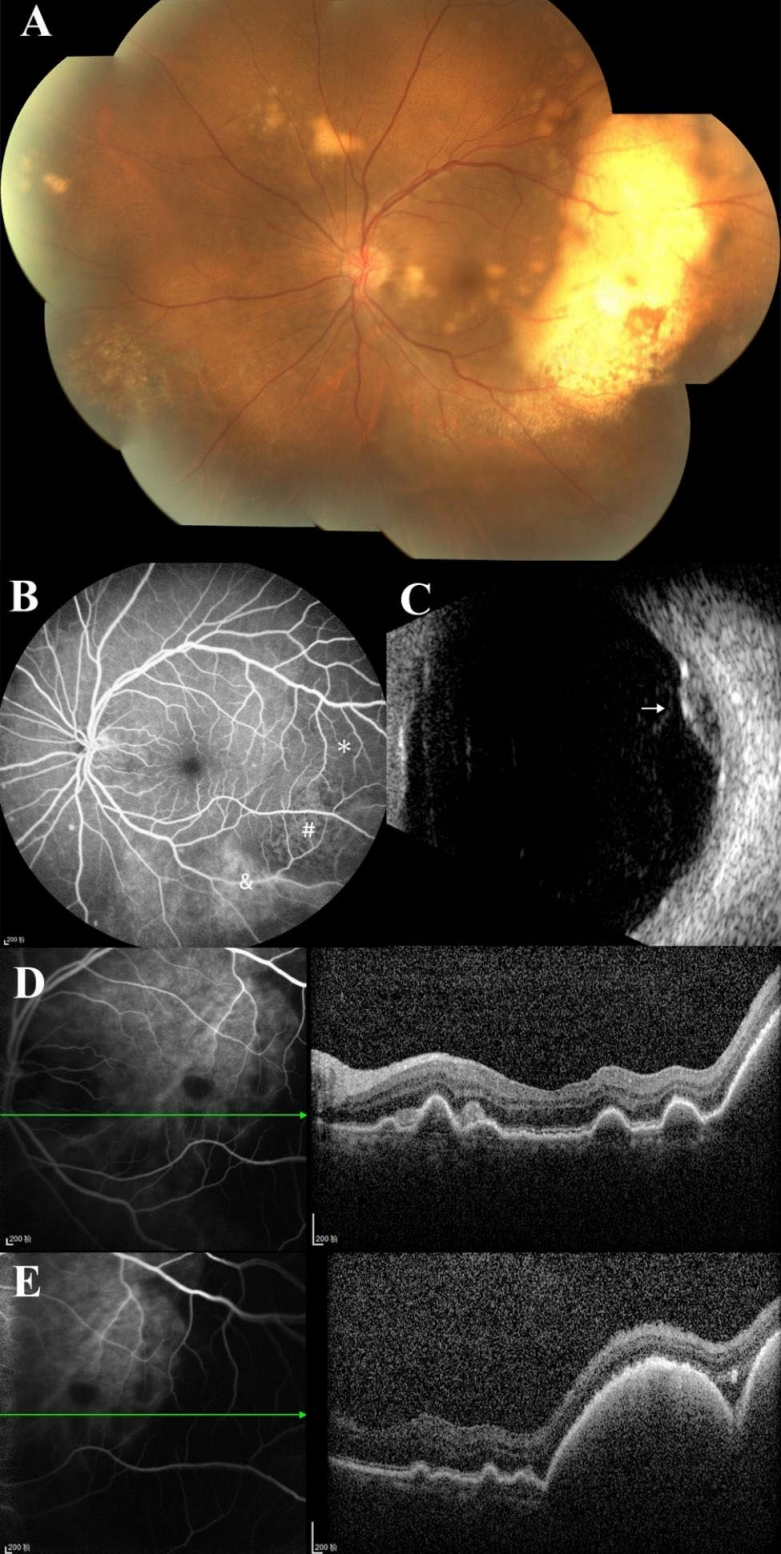
**(A)**Fundus examination showed an elevated yellowish sub-retinal lesion temporal to the macula, and scattered multifocal creamy lesions deep to retina; **(B)**FFA taken at 2 min showed hypo-fluorescence(*), mottled hyper-fluorescence(**#**) and hyper-fluorescence(&) area in the temporal mass(2-7o’clock); the leakage of the inferior part of the lesion(&) increased as the examination progressed; diffuse hyper-fluorescent spots at superior-nasal/inferior-nasal quadrants increased in both size and intensity; **(C)** Ultrasound of the left eye showed a small elevated lesion(arrow); **(D) (E)** OCT of the left eye showed multiple hyper-reflective lesions in the outer retina and beneath RPE at paramacular region, and domed retina temporal to the macula, nodular hyper-reflective signals between the Bruch’s membrane and RPE corresponding to fundus examination

A 71-year-old female presented with a 2-month history of painless blurred vision and an increasing nasal scotoma of her left eye. Best-corrected visual acuity (BCVA) was 20/20 in right eye and 20/30 in left eye. Slit-lamp examination revealed a quiet anterior chamber. Fundus examination of her left eye was notable for mild vitreous opacity, an elevated yellowish subretinal mass temporal to the macula and scattered multifocal creamy lesions deep to retina (Fig. 1A). Fundus fluorescein angiography (FFA) of the left eye showed hypofluorescence with mottled hyperfluorescence in the temporal lesion, leakage of the inferior part of lesion increased as the examination progressed (Fig. 1B). Ultrasound of the left eye showed a small elevated lesion (Fig. 1C) Optical coherence tomography (OCT) of the left eye showed elevated retina and hyper-reflective mass between Bruch’s membrane and RPE temporal to the macula, as well as multifocal nodular hyper-reflective signals in the outer retina and beneath RPE (Fig. 1D/1E). Five years ago, she was diagnosed with gastric MALT lymphoma. Her gastric lymphoma was staged as Ann Arbor IE and Lugano IE. *Helicobacter pylori* in gastric tissue biopsy specimen was negative. She was regularly followed-up without further therapy. As a result, secondary vitreoretinal lymphoma was suspected. Diagnostic PPV was performed. A total of 0.1ml aqueous, 2ml undiluted vitreous and 5ml diluted vitreous sample was sent for analysis. IL-10 level of aqueous sample was 187.7pg/ml (IL-10/IL-6 = 13.5). Cytology, gene rearrangement and flow cytometry for immunophenotyping of the vitreous sample were inconclusive. PET scan and brain MRI were unremarkable. IL-10 level of cerebrospinal fluid was normal. Watchful waiting was decided after discussion with the patient about the potential risk and benefits of further biopsy or intravitreous chemotherapy.

**Fig. 2:**
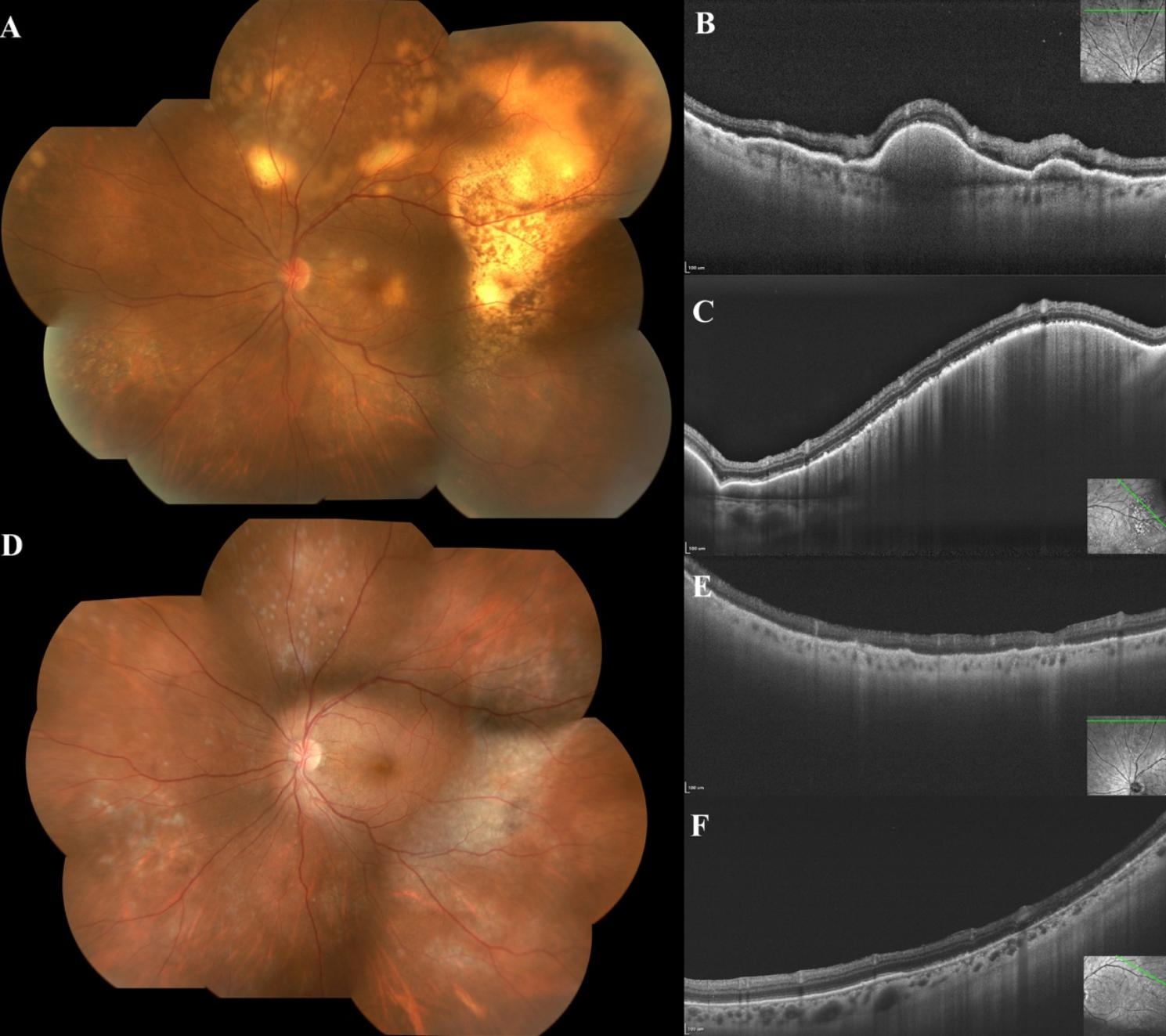
**(A)** Three weeks after PPV, the mass temporal to macula became smaller in size but more lesions at upper quadrant was detected; **(B)(C)** OCT scan showed nodular hyper-reflective signals beneath RPE at upper quadrant. and temporal quadrant; **(D)** Six months after presentation, lymphomatous lesions regressed with residual retinal pigmentation; **(E)(F)** OCT showed disappearance of nodular hyper-reflective signals, and remaining disruption of the photoreceptor/RPE layer

She was followed up regularly. Three weeks after vitrectomy, the height of the temporal mass became lower, but more creamy lesions were observed at upper quadrant (Fig. 2A), corresponding with new nodular hyper-reflective lesions on OCT (Fig. 2B). The hyper-reflective mass temporal to the macula became smaller in size (Fig. 2C). During the following visits, her lymphomatous lesions regressed gradually spontaneously (see supplementary figure). Six months after her presentation, her fundus revealed a residual area of pigmentation overlying the previous site of the subretinal lesions (Fig. 2D). OCT exhibited disruption of the photoreceptor/RPE layer, all nodular hyper-reflective signals disappeared (Fig. 2E/2F). Her final BCVA achieved 20/20 and aqueous IL-10 declined to 64.3pg/ml (IL-10/IL-6 = 2.9).

## Discussion

MALT lymphoma occurs at different extranodal sites, including the stomach (70%), lung (14%), ocular adnexa (12%), thyroid (4%) [5]. Intraocular MALT lymphomas are usually uveal lymphoma and most cases reported in the literature are of conjunctival MALT lymphoma with intraocular extension [3]. Gastric MALT lymphoma is a very indolent subtype. However, our case shows that extension to the extra-gastrointestinal organs does occur. Secondary intraocular involvement by systemic MALT lymphoma is an extremely rare condition. It primarily affects the uveal tissue because of the abundance of blood supply [6]. We reported a case of gastric MALT lymphoma with secondary vitreoretinal involvement and an unexpected good outcome. This case report presented an atypical presentation of gastric MALT lymphoma that has not been previously reported in the literature. This phenomenon reminds us not to underestimate indolent lymphoma. Regular fundus screening is necessary in patients with lymphoma. The novel reaction to biopsy might provide new insights into the future research and treatment of lymphoma.

The definitive mechanism of regression remains unclear, prevailing thoughts are that the patient’s immune system against cancer is activated as a result of physical trauma caused by biopsy or infections [7, 8]. Some cases of regression could be attributed to *Helicobacter pylori* infection, which activating immune response against the lymphoma cell [8]. Several authors have reported abundant CD8 + T lymphocytes infiltrating into tissue of lymphoma with evidence of SR [4, 9]. It is believed that these cytotoxic T cells can attack and induce apoptosis of tumor cells.

However, it has also been reported that PPV-induced T-cell inflammation may only induce temporary regression of B-cell lymphoma [10, 11]. These patients have a high risk of relapse and need continuous monitoring.

## Conclusion

We presented a rare case of a gastric MALT lymphoma patient with intraocular involvement who experienced spontaneous regression after diagnostic PPV without chemotherapy. Multimodal imaging, as well as aqueous IL-10, plays an essential role in diagnosing and monitoring of the disease.

## Electronic supplementary material

Below is the link to the electronic supplementary material.

Supplementary Material 1

Supplementary Material 2

## Data Availability

All data generated or analysed during this study are included in this published article.
