# Supplementary material for: Spontaneous regression of secondary vitreoretinal lymphoma after diagnostic vitrectomy: case report
Source: BMC Ophthalmol. 2023 May 17;23:222. doi: 10.1186/s12886-023-02967-5 (PMC10193612; doi:10.1186/s12886-023-02967-5)
Supplement: Supplementary file 2 — Supplementary Material 2 [file 12886_2023_2967_MOESM2_ESM.docx]

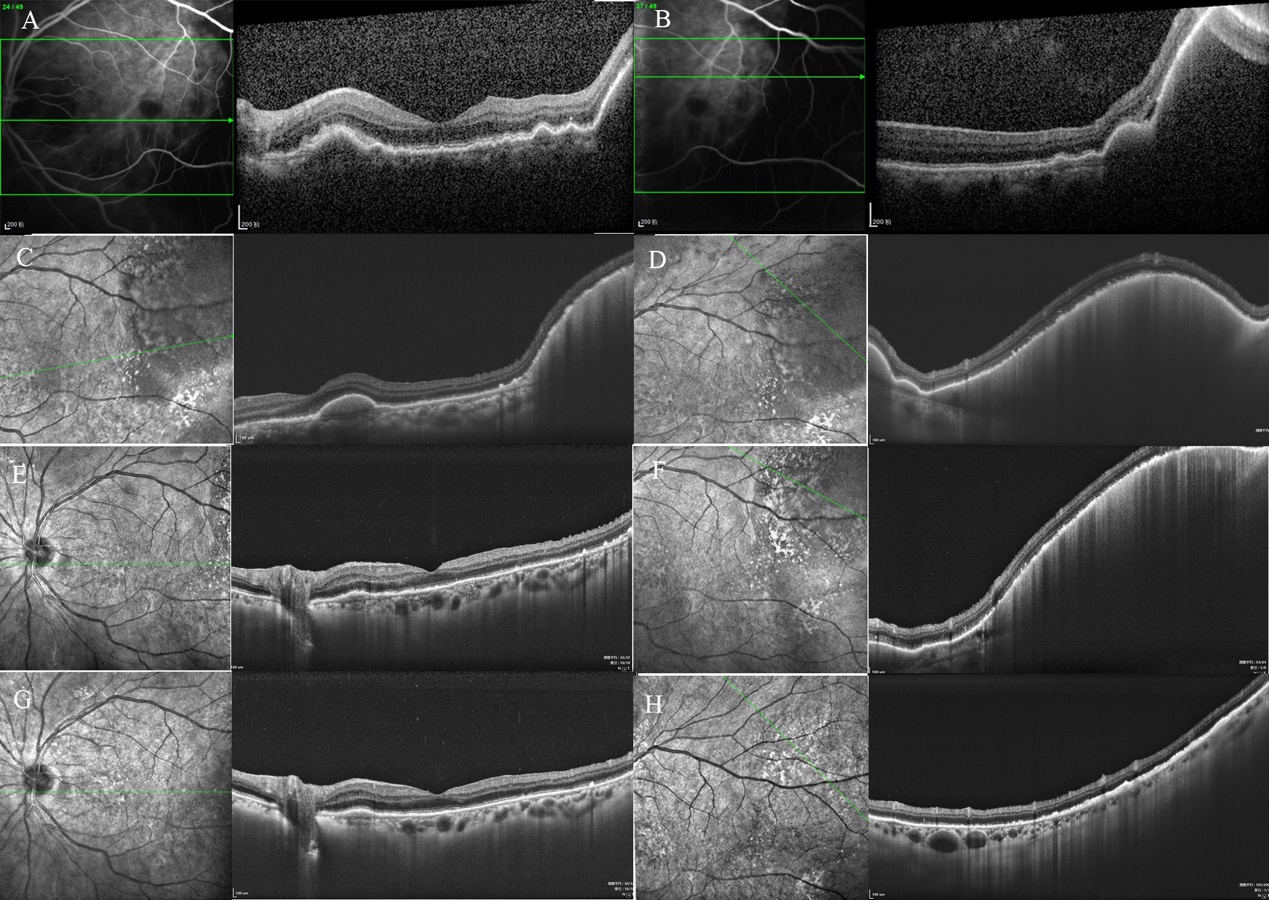


Supplementary figure: OCT scans obtained before (A, B) and three weeks (C, D)/five weeks (E, F)/ two month (G, H) after diagnostic vitrectomy. The scanning lines were not exactly same. However, we can see the hyper-reflective lesions became smaller in size and regressed gradually.
